# Condensed ferric dimers for green photocatalytic synthesis of nylon precursors†
†Electronic supplementary information (ESI) available: Additional data (Fig. S1–S13) and references, and appendix. See DOI: 10.1039/c9sc01253b


**DOI:** 10.1039/c9sc01253b

**Published:** 2019-06-12

**Authors:** Yusuke Ide, Satoshi Tominaka, Yumi Yoneno, Kenji Komaguchi, Toshiaki Takei, Hidechika Nishida, Nao Tsunoji, Akihiko Machida, Tsuneji Sano

**Affiliations:** a International Center for Materials Nanoarchitectonics (MANA) , National Institute for Materials Science , 1-1 Namiki , Tsukuba , Ibaraki 305-0044 , Japan . Email: IDE.Yusuke@nims.go.jp ; Email: TOMINAKA.Satoshi@nims.go.jp; b Department of Earth Sciences , Waseda University , 1-6-1 Nishiwaseda, Shinjuku-ku , Tokyo 165-8050 , Japan; c Graduate School of Engineering , Department of Applied Chemistry , Hiroshima University , 1-4-1 Kagamiyama , Higashi-Hiroshima 739-8527 , Japan; d Synchrotron Radiation Research Center , National Institutes for Quantum and Radiological Science and Technology , 1-1-1, Kouto, Sayo-cho, Sayo-gun, Hyogo 679-5148 , Japan

## Abstract

Although iron oxides have been extensively studied as photocatalysts because of their abundance and environmental compatibility, their performance is notoriously low due to factors such as low photoinduced charge-separation efficiency. Iron oxides, thus, must be modified with expensive and/or toxic materials to attain higher performances, which devalues their appeal as sustainable materials. Here, we design an iron oxide exhibiting an unprecedentedly high photocatalytic performance unrealized by previous photocatalysts such as TiO_2_ for reactions including the selective oxidation of cyclohexane to industrial nylon precursors. The iron oxide photocatalyst consists of ferric dimers, otherwise extremely unstable, formed *via* etching of Fe and O sites from ferric oxide nanoparticles immobilized within porous silica. We demonstrate a remarkably high photoinduced charge-separation efficiency (long lifetime of active species) of the ferric dimers due to their electronic structure and the potential of this supported photocatalyst for many more reactions.

## Abstract 2

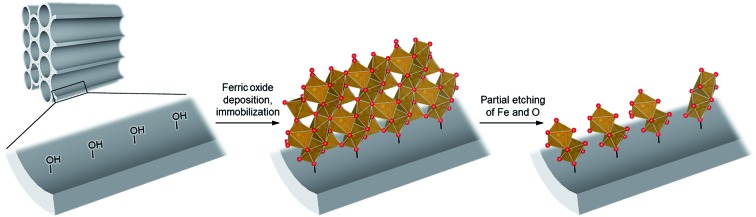
In contrast to usual iron oxides, molecularly small iron oxides synthesized and stabilized on a porous silica scaffold exhibited an unprecedentedly high photocatalytic performance unrealized by previous photocatalysts like TiO_2_.

## Introduction

Liquid-phase aerobic oxidation of cyclohexane is crucially important for the production of cyclohexanone and cyclohexanol (the so-called KA-oil), which are the main industrial precursors of nylon polymers.^1^ KA-oil is currently produced through the aerobic oxidation of cyclohexane using cobalt-based homogeneous catalysts under harsh conditions, at 415–435 K under 1–1.5 MPa. The product, KA-oil, is likely to be over-oxidized, and thus the conversion must be kept at a low rate of 3–5% to prevent over-oxidation, giving 75–85% selectivity of KA-oil.^1^ A number of attempts have thus been made to develop effective catalytic systems for partial cyclohexane oxidation, but they still require the use of hazardous and expensive reagents and materials under harsh conditions.^1^ Solid photocatalysis using solar energy (clean and available in an unlimited supply) and O_2_ (an inexpensive and abundant oxidant) has been considered as a promising alternative, but even typical photocatalysts, such as TiO_2_, often show extremely low yield and selectivity for KA-oil because they easily over-oxidize the products into undesired byproducts, such as CO_2_, due to the presence of highly oxidizing radical species.^2^,^3^ The relatively low conversion compared to that obtained for thermal catalysis has also hampered the practical use of photocatalysis.^4^–^6^ We confirmed this using one of the most active commercial TiO_2_ photocatalysts, P25, which showed up to 0.9% conversion and low selectivity (84%) of KA-oil (Table 1).

**Table 1:** Cyclohexane oxidation with molecular O_2_ using different photocatalysts under irradiation with a solar simulator^*a*^

Photocatalysts	Yield (μmol)			Conversion^*b*^ (%)	Selectivity^*c*^ (%)
	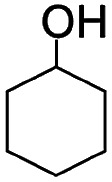	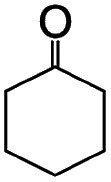	CO_2_		
TiO_2_	21	21	70	0.4	78
TiO_2_^*d*^	18	85	120	0.9	84
TiO_2_ (HCl)^*e*^	10	23	14	0.3	93
α-Fe_2_O_3_	0	0	0	0	—
β-FeOOH	0.2	0.2	0	0.003	>99
FO/SBA	5	7	12	0.1	95
AFO/SBA	67	70	16	1.0	98
AFO/SBA^*d*^	198	377	19	4.1	99
AFO/SBA^*d*^ ^,^^*f*^	191	383	20	4.1	99

^*a*^Conditions: photocatalyst, 30 mg; acetonitrile solution (20 mL) containing cyclohexane, CH (14 mmol); O_2_, 100 kPa; solar simulator irradiation (*λ* > 300 nm, 1000 W m^–2^), 24 h.

^*b*^Determined as 
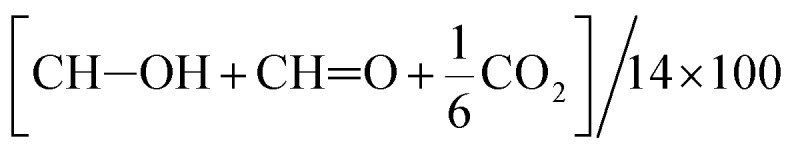
 because oxidized products other than these 3 products were scarcely detected.

^*c*^Determined as 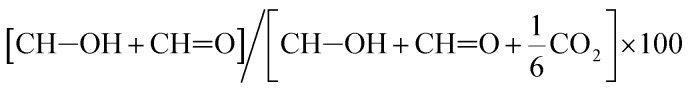.

^*d*^Optimized conditions: photocatalyst, 60 mg; O_2_, 300 kPa; solar simulator irradiation, 48 h.

^*e*^Treated with HCl solution (0.2 M) before using.

^*f*^Re-used after the 1^st^ cycle. No Fe leaching was detected after the 1^st^ cycle.

The development of inexpensive and high-performance photocatalysts (or photoelectrochemical catalysts) is of great current interest not only for KA-oil production, but also for efficient conversion of solar energy into chemical and electric energy. Iron(oxyhydr)oxides such as hematite (α-Fe_2_O_3_) stand out among many candidate materials, including TiO_2_, because iron is by mass the most common element on Earth and also environmentally compatible, and iron oxides have favorable band gaps to absorb visible light, which comprises the majority of incident sunlight.^7^,^8^ However, their performance is notoriously low due to factors such as the short lifetime of the photogenerated charge carriers probably attributable to the electronic band structure.^9^ For example, well-studied iron oxide photocatalysts, such as α-Fe_2_O_3_ and β-FeOOH nanoparticles, were almost inactive in partial cyclohexane oxidation (Table 1). Therefore, tremendous effort has been directed at modifying the electronic structures and performances of iron oxides *via* their hybridization with other materials and heteroelement doping.^10^–^13^ Precious metals and relatively toxic materials are often used for these modifications, which reduces their value as low-cost, environmentally friendly materials. Thus, there is interest in developing high-performance photocatalysts that use only iron oxides and other earth-abundant materials.

Here, we present an iron oxide material with an unprecedented photocatalytic performance for partial cyclohexane oxidation. Molecular ferric species, such as ferric dimers, have a larger energy gap (band gap), and we expect them to display high photocatalytic activity, but they are otherwise extremely unstable.^14^–^16^ They are generally formed by the hydrolysis and condensation route *via* neutralization of a highly acidic solution containing ferric monomers, and larger molecules including ferric dimers are unstable.^16^ Biomolecules such as ferritin utilize ferric sites for catalyzing reactions, where the ferric centers are protected by organic species.^17^ Such protected ferric molecules have also been artificially synthesized.^18^–^21^ Loading on supports such as porous silica is a common strategy for stabilizing otherwise unstable molecular catalysts,^22^ but synthesis of naked ferric molecules stably supported on silica was difficult.^23^ Our material consists of ferric dimers, which are otherwise extremely unstable but stabilized in a porous silica scaffold as revealed below.

**Fig. 1:**
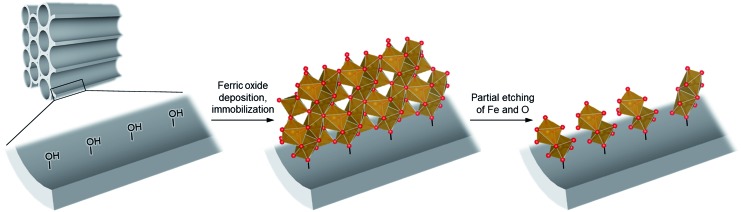
Schematic illustrating the design of molecularly small iron oxides supported within a porous silica. An iron source, Fe(acac)_3_, can graft and condense on the surface hydroxy groups *via* solvothermal reaction to form ferric oxide (ferrihydrite) nanoparticles within the pores. Upon acid treatment, some Fe and O are etched and the strongly immobilized molecularly small iron oxide species remains on the pore walls. Color coding: red = O, brown = Fe.

To achieve the synthesis, we first synthesize ferric oxide nanoparticles deposited and immobilized within a porous silica (SBA-15, pore diameter of *ca.* 9 nm), referred to as “FO/SBA”, *via* a solvothermal reaction of a solution of iron(iii)acetylacetonate, Fe(acac)_3_.^6^ Then, the material is treated with acid to dissolve most of the oxides, but the immobilized ferric species remains (Fig. 1). This synthesis route enables us to access molecularly small iron oxide species, and the loading within SBA-15 stabilizes the dimers *via* chemical bonds so that they can be controlled and utilized in heterogeneous photocatalysis. This product is named “AFO/SBA”.

The photocatalytic performance of AFO/SBA is significantly higher than that of other photocatalysts for KA-oil synthesis: a 4.1% conversion of cyclohexane to cyclohexanone and cyclohexanol, with 99% selectivity was achieved (Table 1). This conversion rate, combined with the selectivity, is by far the highest ever reported for photocatalytic partial cyclohexane oxidation with molecular O_2_ and comparable to that of the current commercial aerobic cyclohexane oxidation using Co-based homogeneous catalysts under harsh conditions.

## Results and discussion

The structures of the products, FO/SBA and AFO/SBA, are fully analyzed as described in the following paragraphs. The as-made product, FO/SBA, had larger α-Fe_2_O_3_ particles deposited outside the silica support (Fig. S1–S3 in the ESI†), but pair distribution function (PDF) analysis indicates that FO/SBA contained nanoparticles (*ca.* 1.6 nm) of a ferric hydroxide, ferrihydrite,^24^,^25^ deposited within SBA-15 (ferrihydrite : α-Fe_2_O_3_ = 67 : 33 by mass, Fig. S3a†). This biphasic state is reasonable for ferrihydrite, because ferrihydrite tends to form α-Fe_2_O_3_ during aging.^25^ Note that a fresh sample immediately after the preparation was composed of pure ferrihydrite within SBA-15 (Fig. S3c and S3d†), suggesting that α-Fe_2_O_3_ was formed during aging (we speculate that solvents trapped within the mesopores played a role in enhancing the formation of α-Fe_2_O_3_, and more systematic investigations are under consideration). A Mössbauer spectrum confirms the Fe^3+^ states and corresponds to typical ferrihydrite samples reported previously (Fig. S4†). Because FO/SBA showed only a moderate conversion and selectivity for the partially oxidized products (Table 1), ferrihydrite and the ordered intermediate are barely active in the reaction (note that α-Fe_2_O_3_ is not active as mentioned above).

**Fig. 2:**
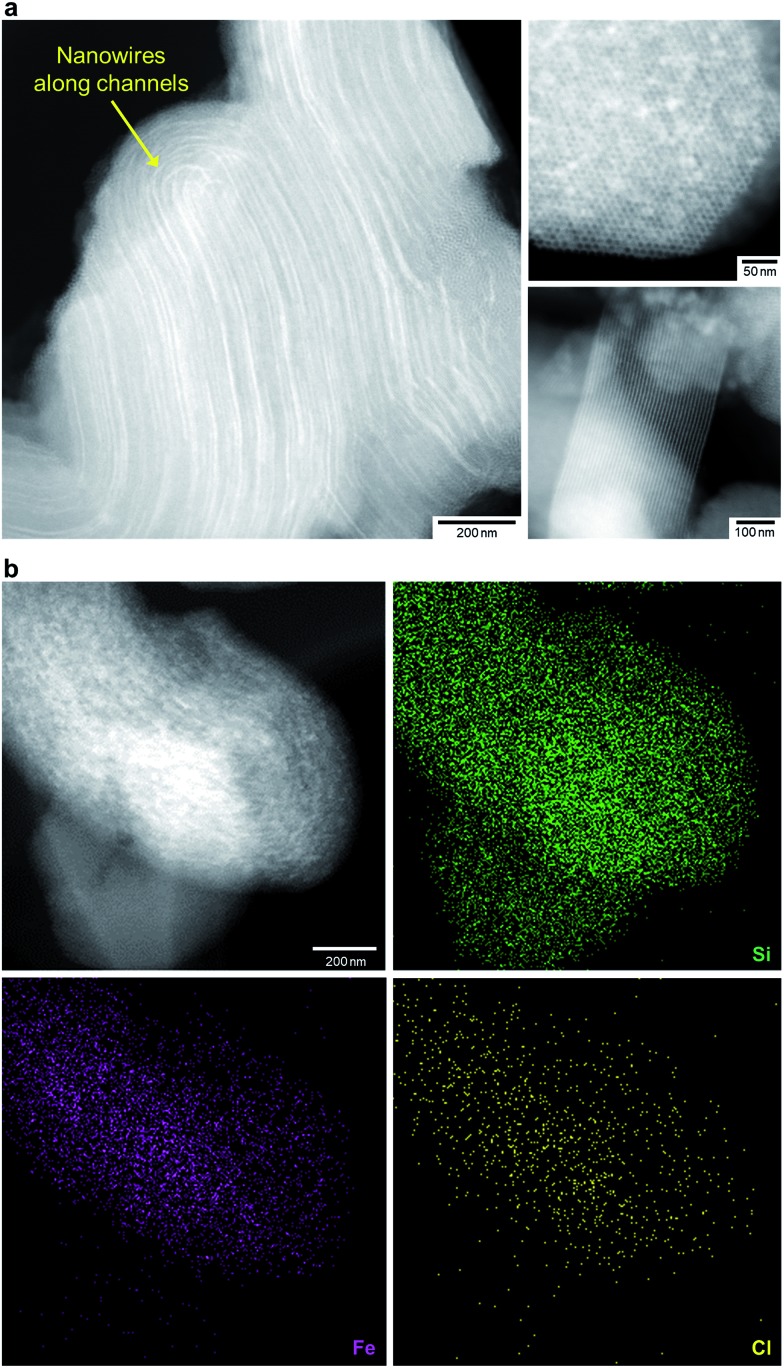
Morphology of AFO/SBA. (a) HAADF-STEM images of AFO/SBA. Many nanowires of iron species are located within the channels of SBA-15 particles (the left and upper right panels), while SBA-15 particles without iron species also exist (the lower right panel). Therefore, the iron loading is only 2.0 wt% on average. (b) The HAADF-STEM image (the upper left panel) and EDX elemental map of AFO/SBA confirm the presence of iron within the SBA-15 particles. Cl was also detected.

We treated FO/SBA with an HCl solution, which decreased the amount of loaded iron from 8.2 to 2.0 wt% (determined by elemental analysis using inductively coupled plasma measurements). No change in the structure of SBA-15 was observed upon solvothermal reaction and acid treatment (Fig. S5†), but the broad scattering intensities of the X-ray diffraction (XRD) pattern of AFO/SBA indicate the presence of a noncrystalline product as well as the remaining α-Fe_2_O_3_ crystals (Fig. S2 and S6†). Note that the XRD pattern was obtained by subtracting the SBA-15 signal (Fig. S2c†) and shows the structural information for the ferric compound only. N_2_ adsorption/desorption isotherms and structural parameters calculated from the isotherms suggest that small iron species in AFO/SBA exist on the pore walls, rather than plugging the pores (Fig. S5†). High-angle annular dark-field (HAADF) images recorded by scanning transmission electron microscopy (STEM) and energy dispersive X-ray (EDX) elemental maps reveal that AFO/SBA contains densely dispersed iron species in the porous silica channels (Fig. 2). These facts suggest the preservation of immobilized noncrystalline iron oxide species on the inner walls of SBA-15.

**Fig. 3:**
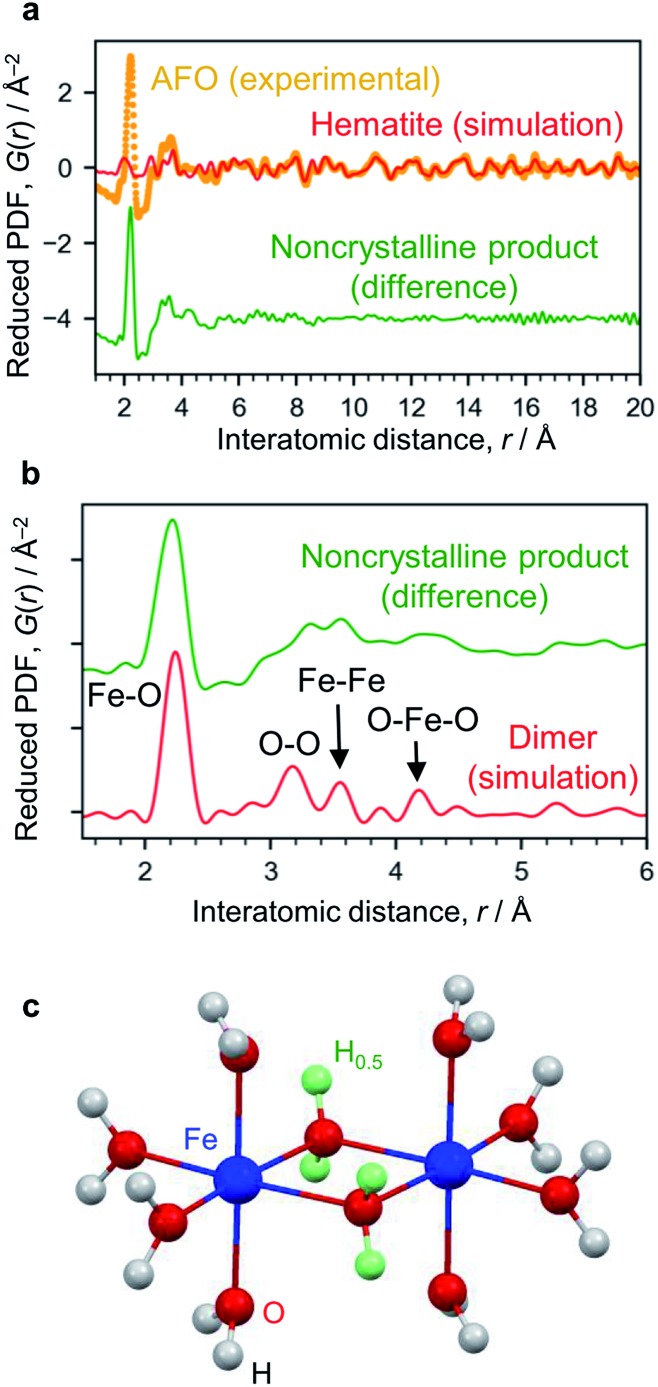
Structure analysis of AFO/SBA. (a) PDF analysis. The experimentally obtained PDF of AFO/SBA (yellow dots) fitted with the hematite structure model in the range of >6 Å (red curve). The difference between the experimental data and the simulated one is shown with a shift for clarity (green curve). (b) Enlarged PDF difference shown in (a). The red curve shown is simulated based on the dimer model shown in (c). (c) Dihydroxo-bridged ferric dimer structure (*D*_2h_, *mmm*). The green spheres show H atoms with an occupancy of 0.5, and were used for calculating the averaged states of the OH groups with lone pairs forming hydrogen bonds. More simulation results are available in ESI Fig. S9.†

The X-ray absorption spectrum (Fig. S7†) and Mössbauer spectrum (Fig. S4†) indicate the Fe^3+^ state. The AFO/SBA contains Fe, Cl and O (Cl/Fe molar ratio = 0.82) as well as Si species from the SBA-15 support (the Cl is thought to originate from residual HCl; however, this scarcely affects the photocatalytic reaction, as described below). To analyze structural details of the noncrystalline phase, the XRD pattern was converted into PDF, which clarified the distances of atomic pairs and their density in the iron oxide species. The PDF in the range of >6 Å can be simulated by the α-Fe_2_O_3_ structure (Fig. 3a and S6†), and the residual PDF reflects the structure of the noncrystalline product. The noncrystalline phase is a major phase because 91% of the 1^st^ PDF peak assigned to the Fe–O nearest neighbors belongs to this (roughly, 91% of Fe ions belong to the noncrystalline phase).

The 1^st^ PDF peak located at approximately 2.2 Å is assigned to Fe–O (Fig. 3b), rather than Fe–Cl, because this distance is shorter than what is usually observed for Fe–Cl (∼2.5 Å) and no Fe–Cl vibration was observed in the infrared spectrum (Fig. S8†). As this distance is longer than that usually observed in iron oxide crystals, *i.e.*, 1.9–2.05 Å,^26^ this 1^st^ peak is assigned to elongated Fe–O bonds. The peaks at ∼3.1 Å and ∼4.3 Å are assigned to the O–O distances of the nearest neighbors and second nearest neighbors in the FeO_6_ octahedra. There are more peaks at 3.32 Å and 3.57 Å, which are typical for the Fe–Fe bond distance, suggesting the formation of condensed structures such as dimers. We carried out theoretical modeling of this system to validate the experimental results, and optimized their structures using quantum chemical calculations (Fig. S9†). The peaks for cationic dihydroxo-bridged ferric dimers, [(H_2_O)_4_Fe(OH)_2_Fe(H_2_O)_4_]^4+^, resemble the experimentally obtained PDF peaks well (Fig. 3c, and S9b and S9c†).

The formation of such ferric dimers was also confirmed by UV-vis spectroscopy (Fig. S8†). AFO/SBA showed an absorption onset (which correlates with the band gap energy) of approximately 420 nm, which is significantly shorter than that of FO/SBA and typical iron oxides (>600 nm). This result is due to the so-called quantum size effect of small molecular ferric dimers. This effect has been extensively investigated for TiO_2_ photocatalysts: the electronic properties dramatically change depending on their structure, from an extended-semiconducting structure to nanoparticles and molecular-sized species. For example, molecular-sized TiO_2_ has a larger energy gap (band gap) than semiconductor TiO_2_.^27^

**Fig. 4:**
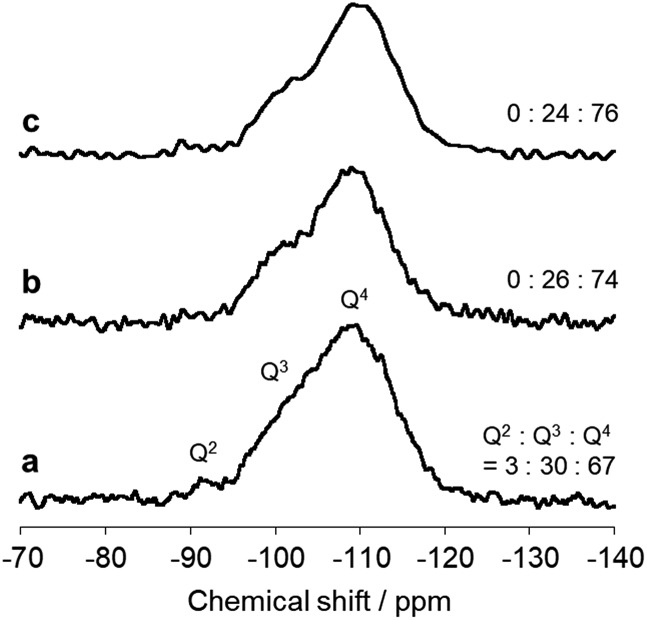
Solid state ^29^Si NMR spectra of (a) SBA-15, (b) FO/SBA and (c) AFO/SBA. The integral (*Q*^2^ + *Q*^3^)/(*Q*^2^ + *Q*^3^ + *Q*^4^) signal ratio is shown on the right.

The immobilized state of ferric dimers was clarified. Solid-state ^29^Si NMR data (Fig. 4) show that the integral (*Q*^2^ + *Q*^3^)/(*Q*^2^ + *Q*^3^ + *Q*^4^) signal ratio, which reflects the amount of surface Si–OH, of AFO/SBA is 0.24; this is identical to that in FO/SBA and significantly smaller than that in SBA-15 (0.33). It was reported that grafting and condensation of Fe(acac)_3_ onto hydroxy groups on oxide surfaces occurred under similar solvothermal conditions to those reported here,^6^ and thus, the decrease in Si–OH indicates that ferric dimers were, at least in part, grafted onto the surface *via* Fe–O–Si bonds (considering the amounts of supported Fe (2.0 wt% corresponds to 0.18 mmol g^–1^ as dimers) and Si–OH of SBA-15 (3.5 mmol g^–1^),^28^ almost all the ferric dimers are grafted). It is reasonable to consider that the formation of these bonds prevents elution of all the deposited ferrihydrite from SBA-15.

The effect of the residual Cl (Experimental section) in AFO/SBA on the photocatalytic activity was investigated, because chlorine radicals have been reported to promote the photocatalytic oxidation of organic compounds with semiconductors.^29^,^30^ We treated P25 with HCl and investigated the photocatalytic activity, but the conversion was scarcely modified (Table 1). Furthermore, AFO/SBA did not have a chlorinated surface (Fig. S8†) in contrast to the chlorine radical-mediated photocatalytic systems.^29^,^30^ Considering that a similar photocatalytic activity was obtained when FO/SBA was treated with other acids, such as HNO_3_ (Experimental section), the high activity of AFO/SBA must originate from the intrinsic activity of the ferric dimers.

We considered possible factors for the high photocatalytic activity of AFO/SBA. The mechanism for the oxidation of organic compounds by semiconductor and molecular sized photocatalysts (*e.g.*, Ti-containing metallosilicates, in which isolated Ti^4+^ atoms are substituted with Si^4+^ atoms in the silica matrixes and coordinated tetrahedrally with O atoms^27^) with O_2_ often involves the capture of photoexcited electrons with O_2_ to produce a relatively stable superoxide radical anion (O_2_^–^) and retard electron–hole recombination (that is, improve charge separation efficiency).^6^,^31^,^32^ Here, we monitored the formation of O_2_^–^*via* electron spin resonance (ESR) analysis. As shown in Fig. S10,† when irradiated by light in the presence of molecular O_2_, P25 showed well-developed signals due to O_2_^–^ (*g*_*xx*_ = 2.003, *g*_*yy*_ = 2.010, and *g*_*zz*_ = 2.022), indicating the formation of a significant amount of O_2_^–^, whereas conventional iron oxides scarcely generated O_2_^–^. On the other hand, when photoirradiated under identical conditions, AFO/SBA showed approximately ten times the O_2_^–^ yield of P25. Given that typical iron oxides don't have electronic band structures (conduction band potentials) capable of reducing O_2_,^9^ these results demonstrate that the ferric dimers in AFO/SBA have an electronic structure that is completely different from that of typical iron oxides and show a charge separation efficiency that is remarkably higher than even that of P25, which is a benchmark photocatalyst for the oxidation of organic compounds.^33^ Based on the structural similarity between the ferric dimers and molecular sized photocatalysts, we can explore the mechanism for photocatalytic cyclohexane oxidation on AFO/SBA according to a proposed mechanism based on molecular sized photocatalysts.^34^ First, excitation of the photocatalyst produces excited Fe^2+^*via* an electron transfer from the coordinated O^2–^ (in H_2_O) to the center Fe^3+^, and the resulting electrophilic O^–^ reacts with cyclohexane to produce a key intermediate, the cyclohexyl radical (Fig. S11†). This intermediate then reacts with reactive oxygen species, including O_2_^–^, to form the partially oxidized products.

We also investigated the effect of SBA-15 support on the photocatalytic activity of AFO/SBA, because mesoporous silica supports used in the design of photocatalysts have some advantages (*e.g.*, large pores facilitating the transfer of organic substrates).^35^ Here, we tested possible selective adsorption of cyclohexane on AFO/SBA to promote (i) oxidation of cyclohexane and (ii) desorption of the cyclohexanol and cyclohexanone formed and inhibit their over-oxidation. When mixed with a mixture of cyclohexane, cyclohexanol and cyclohexanone in the dark, AFO/SBA effectively adsorbed cyclohexane but did not adsorb the latter two at all (Fig. S12†). This selectivity is mostly attributable to SBA-15 (Fig. S12†), and appears to be enhanced in AFO/SBA probably (i) due to the decrease of surface silanol groups of SBA-15, which may interact with polar groups in the oxidized molecules, and (ii) due to the decrease of pore size (Fig. S5†). This consideration of enhanced molecular sieving effect is reasonable because the polar groups of the oxidized species may interact with each other to form larger associates, which enter the nanopores with difficulty. In contrast, P25 adsorbed all three substrates. This accounts for the easy over-oxidation of the partially oxidized products formed by P25. Accordingly, AFO/SBA can effectively oxidize cyclohexane and promptly release the formed cyclohexanol and cyclohexanone from the active center (ferric dimers within the channels), resulting in the unprecedented effective and selective formation of the partially oxidized products.

We compared the performance of the present AFO/SBA with conventional solid photocatalysts. As an example of state-of-the-art photocatalysts for cyclohexane oxidation, we synthesized SBA-15 containing isolated Ti^4+^ species grafted on the pore surface that are coupled with α-Fe_2_O_3_ nanoparticles (mainly working to lengthen the lifetime of the active Ti species).^6^ This material showed a KA-oil yield of up to 0.43 mmol (equivalent to a turnover number (TON) of 8 based on Ti and Fe contents) and nearly 100% selectivity at 2.3% cyclohexane conversion under similar conditions. It is clear that the present AFO/SBA shows a superior performance (up to 27 TON based on Fe content) compared with the previous photocatalyst.

To show the versatility of this photocatalyst, we investigated another photocatalytic reaction, the oxidation of formic acid in water into CO_2_, which is a representative reaction to test the performance of photocatalysts for organic pollutant removal in environments^33^,^36^,^37^ (Fig. S13a†). Again, AFO/SBA showed considerably higher activity than P25, a benchmark photocatalyst for this reaction,^33^ though α-Fe_2_O_3_ scarcely showed the activity. The high photocatalytic activity of AFO/SBA for this reaction was also explained by the high charge separation efficiency *via* formation of O_2_^–^ by reduction of O_2_ with photoexcited electrons.^9^

We also tested the photocatalytic activity of AFO/SBA for H_2_ evolution from water in the presence of a sacrificial agent, methanol.^33^ As shown in Fig. S13b,† AFO/SBA showed a significant activity for this reaction, whereas α-Fe_2_O_3_ showed no activity. Given that usual ferric oxides don't have an electronic band structure (conduction band potential) capable of reducing H^+^ into H_2_, this result confirms again the unique electric structure of ferric dimers.

## Conclusions

We synthesized a novel green, environmentally compatible photocatalyst, ferric dimers stabilized on mesoporous silica, for producing industrially important KA-oil. Our synthetic strategy was to immobilize ferric oxide nanoparticles on the walls of mesoporous silica, which was partially etched to form condensed ferric dimeric molecules stabilized on the silica surface. The performance of this photocatalytic system (99% selectivity of KA-oil and 4.1% cyclohexane conversion at 48 h-irradiation) is significantly higher than that of other solid photocatalysts. As is often the case for photocatalytic systems, the conversion rate is still lower than that of classical catalysts; for instance, the current aerobic oxidation using a Co-based homogeneous catalyst requires only 40 min to reach 4% cyclohexane conversion with a ∼80% selectivity.^1^ Thus, for practical use, further improvement by optimizing synthetic conditions and reaction conditions (*e.g.*, ferric dimer loading, catalyst amount, and light intensity) is necessary. We also plan to investigate the use of related molecular catalysts as photocatalysts for oxidizing a range of other organic compounds for fine chemical synthesis and environmental purification.

## Experimental section

### Materials synthesis and preparation

SBA-15 was prepared by a literature method.^38^ SBA-15 (720 mg) was mixed with an ethanol/hexane mixed solution (3 : 17 in vol/vol, 120 mL) containing 455 mg of Fe(acac)_3_ (>98.0%, Tokyo Chemical Industry). The nominal Fe loading was estimated as 9.1 wt% ([Fe]/[Fe_2_O_3_ + SBA-15]) if all the added Fe(acac)_3_ would be converted to Fe_2_O_3_. Compared with the value obtained by elemental analysis (8.2 wt%), we found that the yield of Fe species in FO/SBA was very high, >90%. The mixture was placed in a Teflon-lined stainless steel autoclave and heated at 100 °C for 24 h. After the reaction, the solid product was separated by centrifugation (3500 rpm, 20 min) and the supernatant was removed by decantation. The product was dried at 60 °C in air for ∼12 h, obtaining FO/SBA. FO/SBA was added to an ethanol/0.2 M HCl mixture (1 : 1 in vol/vol, 40 mL), and the dispersion was stirred for 20 min. After centrifugation, the supernatant was removed, and the solid was treated likewise twice more. The final product was dried at 60 °C in air for ∼12 h to obtain AFO/SBA. P25 was supplied by Nippon Aerosil and used as received. α-Fe_2_O_3_ and β-FeOOH nanoparticles were prepared according to the literature.^39^,^40^

### Materials characterization

^57^Fe Mössbauer spectra were measured with a conventional transmission geometry. XRD patterns for the lower and higher 2*θ* regions were collected using a Rigaku Rint-2500 and Rigaku SmartLab, respectively. N_2_ adsorption/desorption isotherms were obtained at 196 °C using a MicrotracBel Belmax instrument. Prior to performing the adsorption measurements, the samples were heated at 120 °C for 3 h under vacuum. HAADF-STEM images and EDX elemental mapping were obtained by using a JEOL JEM 2100F instrument. Fourier transform infrared (FTIR) spectra were measured on a Shimadzu IRTracer-100 spectrometer. The amount of Fe in FO/SBA and the amounts of Fe and Cl in AFO/SBA were determined by using inductively coupled plasma (ICP) optical emission spectroscopy (Hitachi 720ES) and ion chromatography (Dionex ICS-1600), respectively, on the dissolved sample. UV-vis diffuse-reflectance spectra were recorded on a Shimadzu UV-3100PC spectrometer. ^29^Si magic-angle spinning (MAS) NMR spectra were recorded at 119.17 MHz on a Varian 600PS solid-state NMR spectrometer using a 6 mm diameter zirconia rotor.

### X-ray PDF measurements

X-ray total scattering data for obtaining pair distribution functions (PDFs) were collected using synchrotron irradiation at BL22XU (*λ* = 0.1774 Å) of SPring-8 with a Varex Imaging XRD1621 flat panel detector with a two second exposure time and 60 min integration time. The energy was absolutely calibrated using CeO_2_ (NIST 674b), and the detector distance was 303.7 mm. The samples were sealed in Hilgenberg quartz capillaries (outer diameter: 2.0 mm). From the integrated intensities of the sample data, only the intensities of the capillary were subtracted. Polarization, oblique incidence with absorption correction for the CsI scintillator layer, and area corrections were performed. The data were then converted into 1D total scattering data using the PIXIA program.^41^ We assumed that correlation of X-ray scattering from SBA-15 and that from the iron oxides was negligible, and thus, the total scattering data from the oxides only were obtained by subtracting the total scattering pattern of SBA-15 from the patterns of the composite samples. After fluorescence and Compton scattering corrections, the data were normalized with the form factor based on the Faber–Ziman formalism calculated using atomic scattering factors with the MaterialsPDF program.^41^ The structure function, *S*(*Q*), was converted into reduced PDFs, *G*(*r*), by Fourier transform in the *Q* range of 1.42–23.7 Å^–1^. *G*(*r*) was analyzed by curve fitting using the PDFgui program.^42^ PDFs for the dimer models were simulated using the PDFgui program for the structural models obtained by DFT geometry optimization. The isotropic atomic displacement parameters of *U* = 0.01 Å^2^ (for H) and 0.001 Å^2^ (for others) were used. The XRD pattern was simulated using the Mercury program.^43^

### X-ray absorption data collection

Fe K-edge X-ray absorption spectroscopy (XAS) was performed at BL5S1, AichiSR at 25 °C in transmission mode using a step-scan setup (1 s per step, 620 steps). The powder samples were mixed with boron nitride powder using a mortar and pestle for 30 min; the mixtures were then pelletized using a hand press. The pellets were sealed in nylon films. The obtained XAS was processed using the Athena program^44^ to calibrate energy using Fe metal foil, remove background intensities, normalize the intensities, and Fourier transform the *k*^2^-weighted spectrum over a *k* range of 1–12 Å^–1^ with the Hanning window function.

### Quantum chemical calculations

The calculations were performed by using density functional theory (DFT) with the CASTEP program. Ultrasoft pseudopotentials and an energy cut-off of 260 eV were used. A plane-wave basis set was used, and the exchange–correlation was treated with the generalized gradient approximation (GGA) by using the Perdew, Burke and Ernzerhof (PBE) exchange–correlation functional. Geometry optimizations of several ferric dimers, as shown in Fig. S9,† were performed.

### Photocatalytic test

Cyclohexane oxidation reactions were carried out by photoirradiation with a solar simulator (*λ* > 300 nm, 1000 W m^–2^ – the so-called 1 SUN) in a closed, stainless-steel container (75 mL) equipped with a Pyrex glass window under shaking. The vessel contained a mixture of sample (30 mg) and an O_2_-bubbled solution (20 mL) of acetonitrile containing cyclohexane (1.5 mL equivalent to 14 mmol) unless mentioned otherwise. CO_2_ and organic compounds were quantitatively analyzed by gas chromatography equipped with a flame ionization detector (GC-FID, Shimadzu GC-2014) and a thermal conductivity detector (GC-TCD, Shimadzu GC-8A). No leaching of Fe species from AFO/SBA was detected by ICP elemental analysis for the supernatant after the reaction. In addition to FO/SBA treated with 0.2 M HCl (AFO/SBA), we tested the photocatalytic activity of FO/SBA treated with HNO_3_ (0.2 M) or H_2_SO_4_ (0.2 M). No significant difference in the photocatalytic activity of the three samples was observed. Oxidative decomposition of formic acid was performed in a Pyrex glass tube (34 mL) as follows: the sample (15 mg) was added in an O_2_-bubbled aqueous solution (5 mL) containing 5 vol% of formic acid, and the suspension was ultrasonicated for 3 min and then irradiated with a solar simulator under stirring. The gas in the container was withdrawn with a gas-tight syringe and quantified using a gas chromatograph with a barrier ionization discharge (BID) detector (Shimadzu BID-2010 Plus). H_2_ evolution was carried out in a similar manner. The sample (15 mg) was dispersed in an aqueous solution (5 mL) containing methanol (1 : 1 in v/v), and the suspension was deaerated by Ar bubbling and then irradiated with a solar simulator in the presence of H_2_PtCl_6_·6H_2_O (Pt per sample = 0.5 wt%).

### ESR analysis

ESR measurements were conducted using a JEOL JES RE-1X spectrometer (X-band). The magnetic field was calibrated, and the radical yields were determined from ESR spectra recorded with a Mn^2+^/MgO marker as an external standard reference. The sample powder (10 mg) was placed in a Suprasil ESR tube (external diameter 5 mm), which was evacuated at 150 °C for 1 h and cooled to room temperature. O_2_ (20 Torr) was introduced into the tube and retained for 10 min. The sample was photoirradiated at room temperature using a 500 W Xe lamp at *λ* > 330 nm for 5 min. The sample was then evacuated for 10 min to remove excess O_2_ and subjected to ESR measurements at –196 °C.

### Adsorption test

SBA-15 (30 mg) was dispersed in a mixed solution (20 mL) containing cyclohexane (14 mmol), cyclohexanone (80 μmol) and cyclohexanol (80 μmol) in acetonitrile, and the dispersion was stirred for 4 h. After separation by filtration, the amounts of the remaining organic compounds in the supernatants were quantified by GC-FID.

## Conflicts of interest

There are no conflicts to declare.

## Supplementary Material

Supplementary information
