# Supplementary material for: Condensed ferric dimers for green photocatalytic synthesis of nylon precursors
Source: Chem Sci. 2019 Jun 12;10(27):6604–11. doi: 10.1039/c9sc01253b (PMC6625416; doi:10.1039/c9sc01253b)
Supplement: Supplementary file 1 [file SC-010-C9SC01253B-s001.pdf]

Electronic Supplementary Material (ESI) for Chemical Science.
This journal is © The Royal Society of Chemistry 2019

ESI for:

Condensed Ferric Dimers for Green Photocatalytic Synthesis of Nylon Precursors

Yusuke Ide[a*†], Satoshi Tominaka[a*†], Yumi Yoneno[b], Kenji Komaguchi[c], Toshiaki Takei[a], Hidechika Nishida[c], Nao Tsunoji[c], Akihiko Machida[d] and Tsuneji Sano[c]

a International Center for Materials Nanoarchitectonics (MANA), National Institute for Materials Science, 1-1 Namiki, Tsukuba, Ibaraki 305-0044, Japan.
E-mail: IDE.Yusuke@nims.go.jp (YI); TOMINAKA.Satoshi@nims.go.jp (ST)
b Department of Earth Sciences, Waseda University, 1-6-1 Nishiwaseda, Shinjuku-ku, Tokyo 165-8050, Japan.
c Graduate School of Engineering, Department of Applied Chemistry, Hiroshima University, 1-4-1 Kagamiyama, Higashi-Hiroshima 739-8527, Japan.
d Synchrotron Radiation Research Center, National Institutes for Quantum and Radiological Science and Technology, 1-1-1, Kouto, Sayo-cho, Sayo-gun, Hyogo 679-5148, Japan.
† These authors contributed equally.

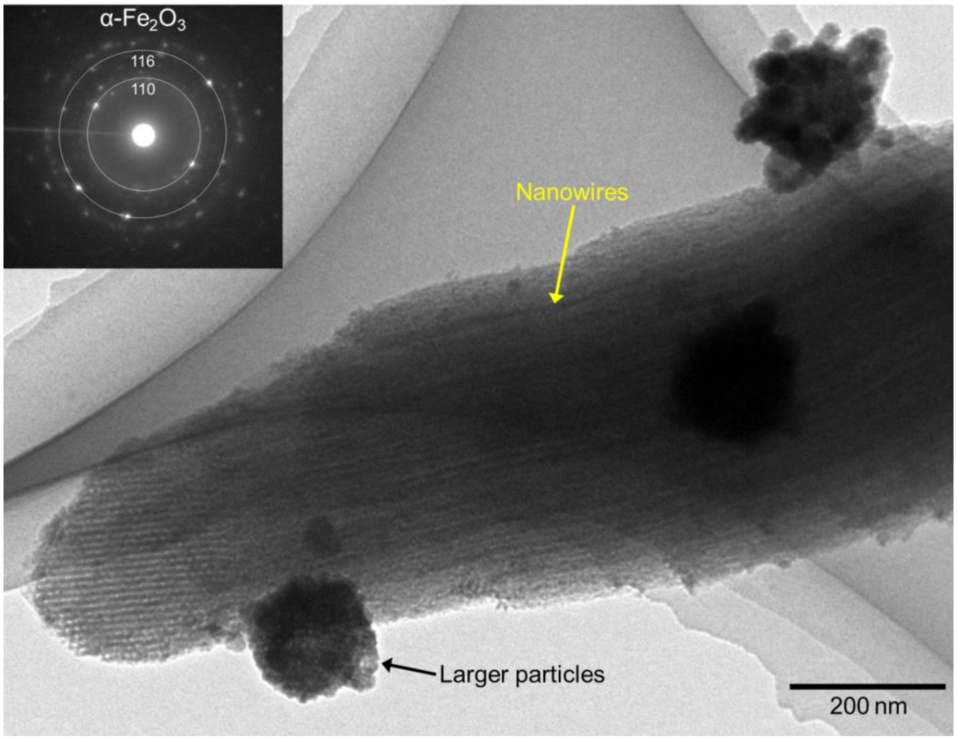

Fig. S1. Morphology of FO/SBA. TEM image of FO/SBA. In addition to nanowire-like particles within the SBA-15 particles, larger particles can be observed on the SBA-15 particles. Inset shows selected area electron diffraction (SAED) pattern taken from latter larger particles, which reveals that these are aggregates of α-Fe_2O_3 nanoparticles.

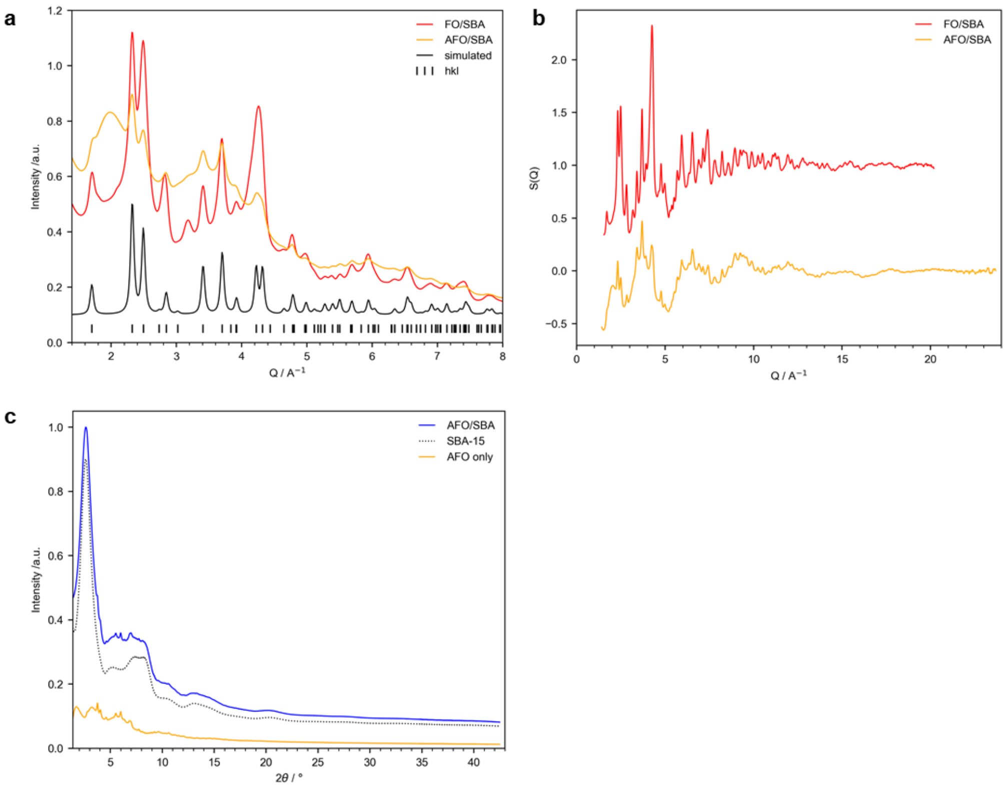

Fig. S2. X-ray total scattering analysis. **a,** Total scattering intensities vs. scattering vector (reciprocal lattice space data). The red and yellow curves show the experimentally obtained data for the samples before (FO/SBA) and after (AFO/SBA) acid treatment, respectively. The scattering intensity from SBA-15 mesoporous silica was subtracted. The black curve and black vertical lines show the simulated pattern using the Mercury program and the hkl positions for hematite (α-Fe_2O_3), respectively. **b,** Total structure function, *S*(*Q*), of the samples before and after the acid treatment. The data for AFO/SBA were shifted by -1.0 for clarity. **c,** Raw total scattering intensities vs. scattering angle before (AFO/SBA) and after (AFO only) subtracting the SBA-15 intensity.

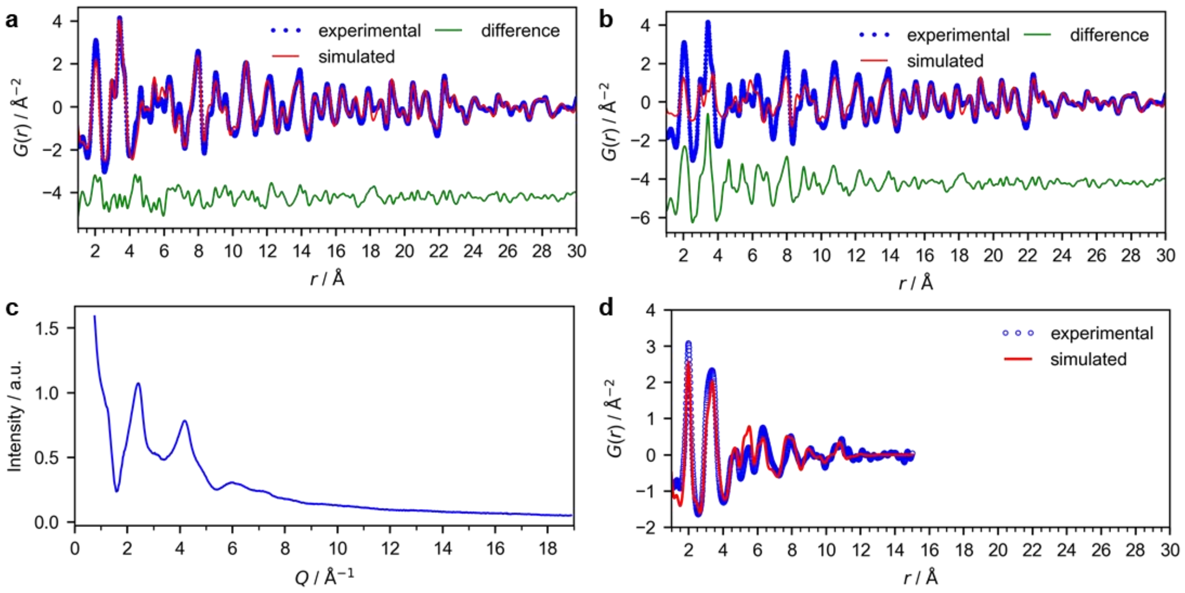

Fig. S3. X-ray PDF analysis of the sample before acid treatment (FO/SBA). a, The PDF obtained by the synchrotron X-ray experiments was fitted with the structural model of hematite and ferrihydrite. The presence of hematite was clearly suggested by the reciprocal space data (**Fig. S2a**). **b,** The contribution of the hematite phase only in the curve fitting. The difference can be assigned to ferrihydrites, as reported in the literature.[2] Note that the contribution of SBA-15 was subtracted as described in the experimental section. **c, d,** A fresh sample immediately after the preparation was composed of pure ferrihydrite. **c,** Total scattering intensity vs. scattering vector (reciprocal lattice space data) obtained by Ag Kα radiation. The scattering intensity from SBA-15 mesoporous silica was subtracted. The data resembles reported diffraction patterns for so-called 2-line ferrihydrite.[2] **d,** Reduced PDF, $G(r)$ (Qmax = 18.9 Å^{-1}, with the revised Lorch function,[3] Δ = 1.0). The experimental data, blue curve, was analyzed by the curve fitting with the reported ferrihydrite model (red curve).[2]

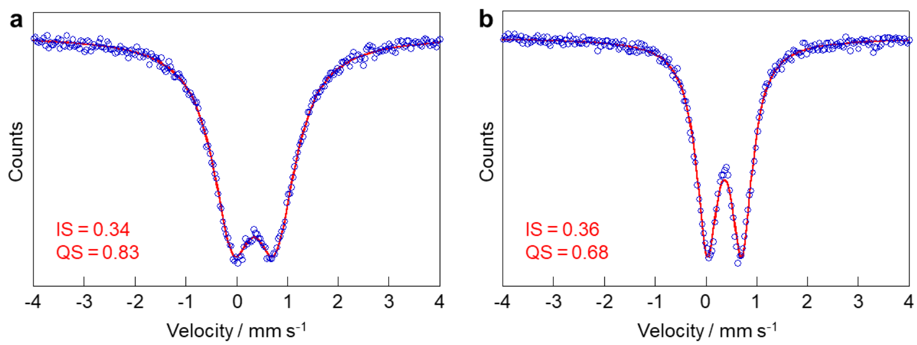

Fig. S4. ^{57}Fe Mössbauer spectra of (a) FO/SBA and (b) AFO/SBA at 300 K. a, FO/SBA has a paramagnetic doublet with an isomer shift (IS) of 0.34 mm s^{-1} and quadrupole splitting (QS) of 0.83 mm s^{-1}. This relatively small IS value is characteristic of Fe^{3+}. The broadness of the peaks means the multiphasic state as described in the main text and is consistent with a literature.[4] **b,** AFO also has paramagnetic doublet with parameters IS 0.36 mm s^{-1} and QS 0.68 mm s^{-1}. The relatively small IS value again confirms the Fe^{3+} state.

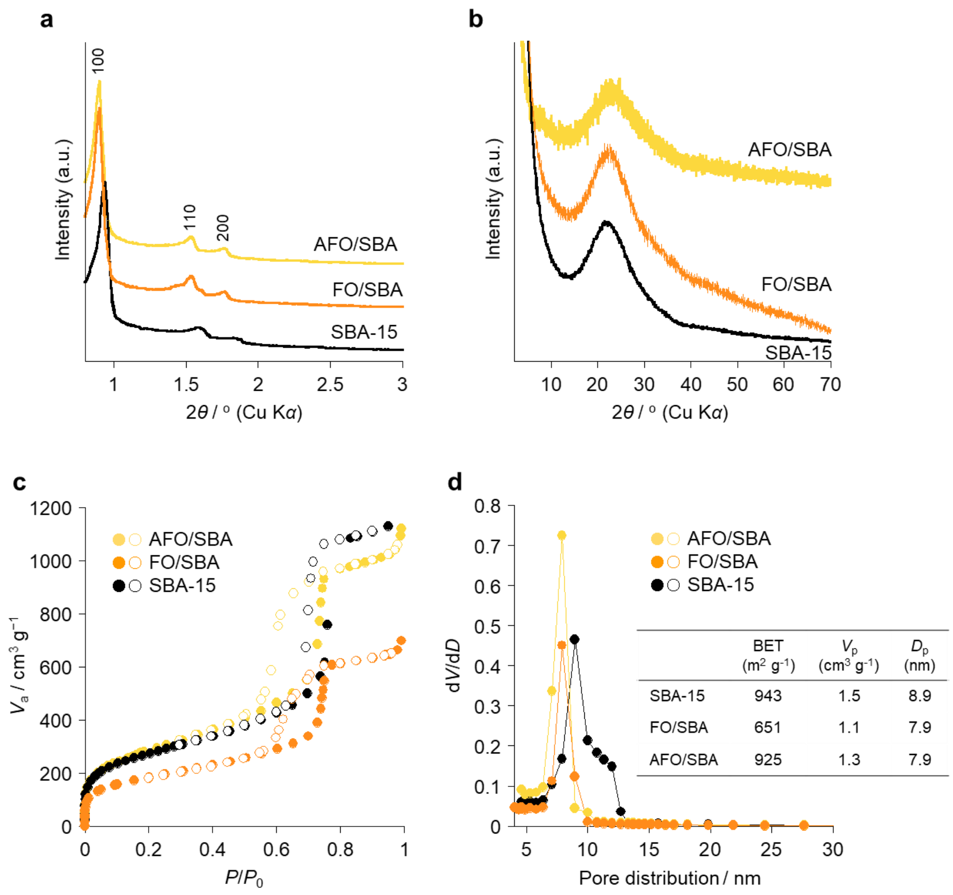

Fig. S5. Mesostructure of AFO/SBA. a, Small-angle powder XRD patterns of SBA-15, FO/SBA and AFO/SBA. SBA-15 shows (100), (110) and (200) reflections, which are typical of hexagonally ordered mesoporous structures, while FO/SBA and AFO/SBA show a profile similar to that of SBA-15. **b,** Wide-angle powder XRD patterns of SBA-15, FO/SBA and AFO/SBA. No diffraction peaks assignable to iron oxides are observed in this laboratory X-ray data for FO/SBA and AFO/SBA, but the synchrotron X-ray data can clarify the presence of hematite and ferrihydrite as well as the non-crystalline molecular species (cf. **Fig. S2**). **c,** N_2 adsorption/desorption isotherms of SBA-15, FO/SBA and AFO/SBA. The all isotherms are type IV, indicating the presence of uniform mesopores. **d,** BJH pore size distributions inserted with textural properties evaluated from the isotherns. The BET surface area, mesopore volume and pore size of AFO/SBA all decreased relative to those of SBA-15. This is ascribable to the iron species immobilized on the pore walls.

However, AFO/SBA still exhibits a relatively high BET surface area and mesopore volume and a narrow pore distribution. On the other hand, the BET surface area, mesopore volume and pore size of FO/SBA largely decreased compared to those of SBA-15, meaning the presence of larger ferric oxides species within the mesopores.

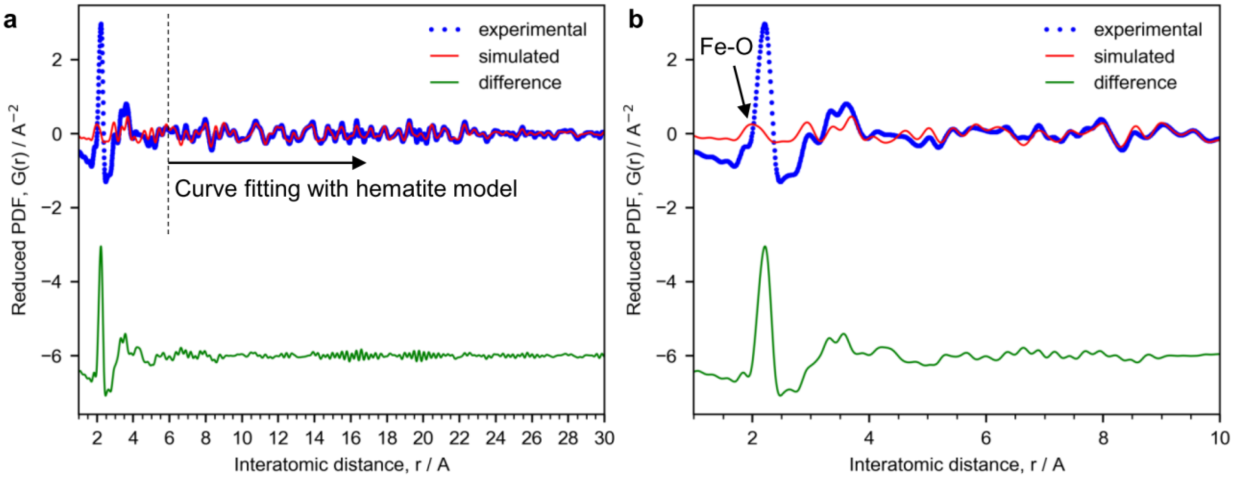

Fig. S6. X-ray PDF analysis of the sample after acid treatment (AFO/SBA). a, PDF in the range from 6 to 30 Å was fitted with the structural model of hematite, whose presence was suggested by the reciprocal space data, and then, the difference in the entire range was obtained by simulation using the obtained hematite structure. Note that the contribution of SBA-15 was subtracted, as described in the experimental section. As shown in panel **'b',** the experimental PDF has a tiny peak at approximately 2.0 Å, which can be simulated reliably with the hematite model.

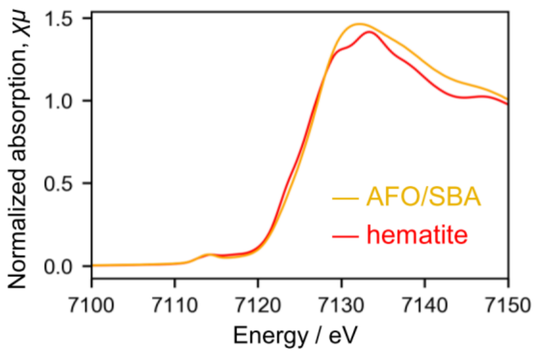

Fig. S7. **X-ray absorption spectra for hematite (red curve) and the AFO/SBA catalyst (yellow curve).**

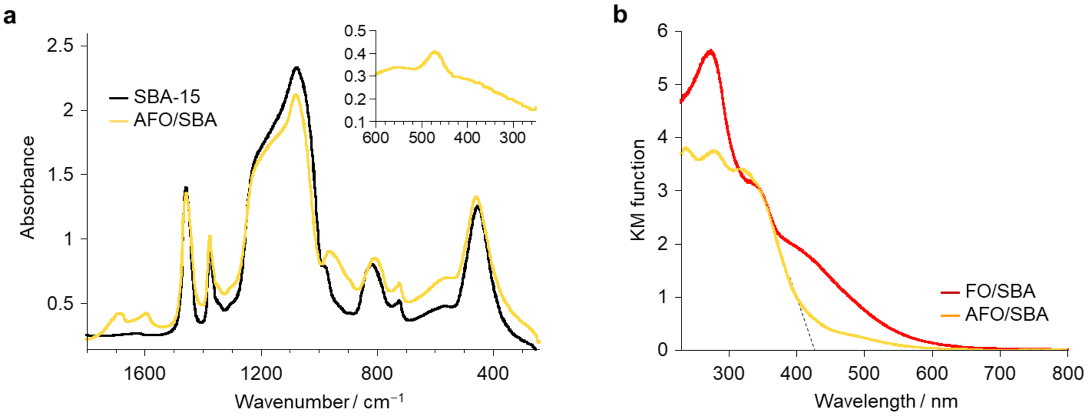

Fig. S8. a, FTIR spectra of SBA-15 and AFO/SBA. The inset shows the difference spectrum. An absorption band at 269 cm^{-1} due to the Fe-Cl stretching vibration[5] was not observed. **b,** UV-vis spectra of FO/SBA and AFO/SBA. The absorption onset associated with the main component in AFO/SBA can be estimated as 420 nm. The absorption at wavelength region longer than 420 nm is assigned to the remaining hematite (α-Fe_2O_3).

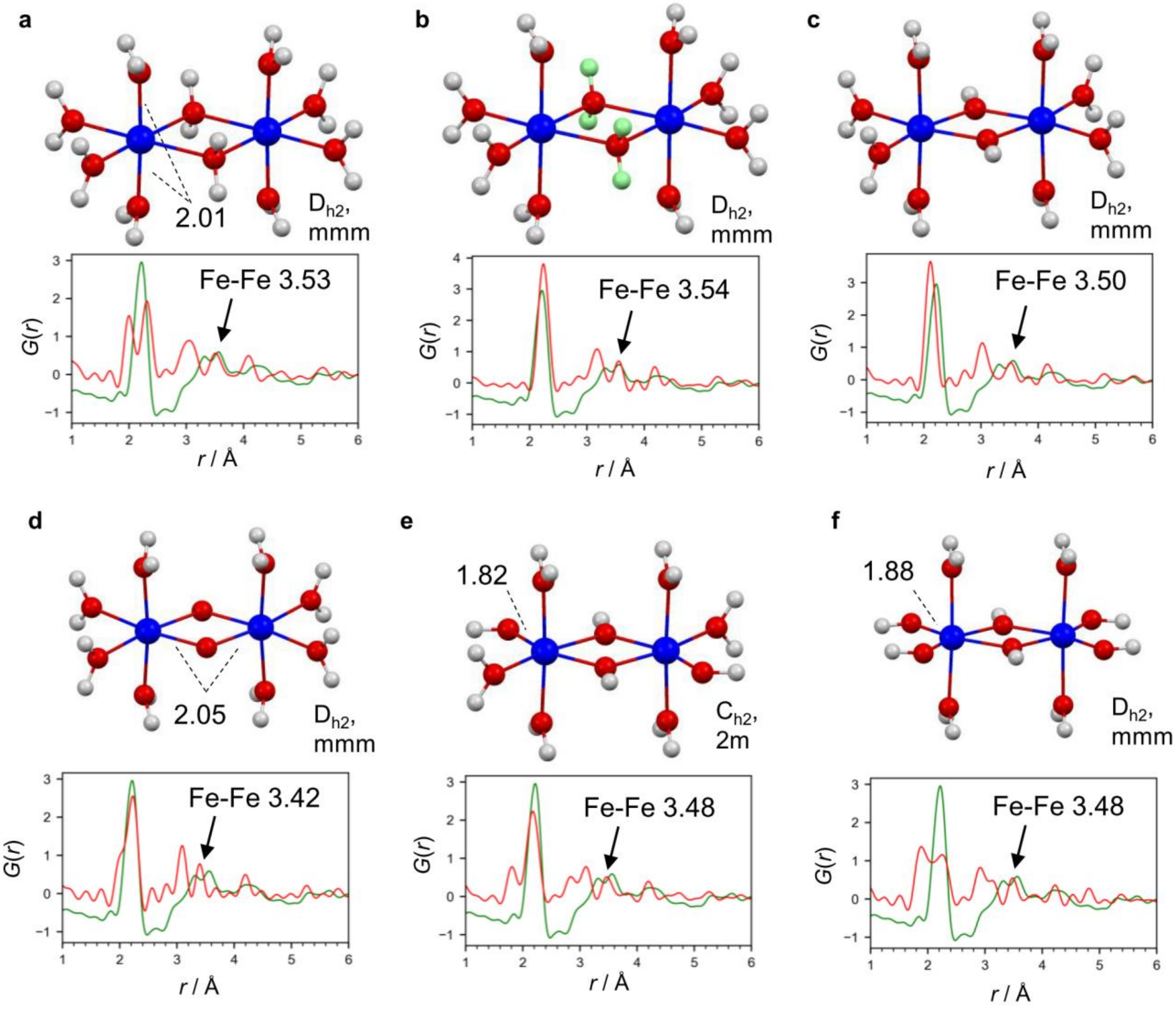

Fig. S9. PDF simulation of ferric dimer structures optimized by quantum chemical calculations. Structure models of iron dimers (top) and simulated PDFs (bottom, red curves). The green curves are the experimental PDFs after subtracting the PDFs simulated for the hematite-structure model. The PDF simulation of a dimer model was performed for a dimer molecule placed in a $10 \times 15 \times 15$ Å^3 unit cell; thus, the data do not simulate density information. This is the cause of the upward shift of the simulated PDF relative to the experimental PDF. The numbers are distances in Å. **a,** $[(H_2O)_4Fe(H_2O)_2Fe(H_2O)_4]^{6+}$, where ferric cations are bridged by two water molecules. **b,** $[(H_2O)_4Fe((H_{0.5})_2O)_2Fe(H_2O)_4]^{4+}$, where ferric cations are bridged by two OH

molecules with a hydrogen-bonding network. The hydroxyl groups are modeled as H_2O molecules with an occupancy of 0.5 for the H atoms. **c,** $[(H_2O)_4Fe(OH)_2Fe(H_2O)_4]^{4+}$, where ferric cations are bridged by two OH^- anions. **d,** $[(H_2O)_4Fe(O)_2Fe(H_2O)_4]^{2+}$, where ferric cations are bridged by two O^{2-} anions. **e,** $[(H_2O)_3(OH)Fe(OH)_2Fe(H_2O)_3(OH)]^{2+}$, where ferric cations are bridged by two OH^- anions. **f,** $[(H_2O)_2(OH)_2Fe(OH)_2Fe(H_2O)_2(OH)_2]$, where ferric cations are bridged by two OH^- anions.

The 1st PDF peak is symmetric and sharp, which suggests the presence of ferric dimers with a relatively high symmetry. These calculations reveal that the Fe-Fe distance of the ferric dimers depends on the number of protons on the bridging oxygen atoms. The dimer with OH in an H_2O-like geometry is the closest representation (**Fig. 3c**). This indicates that the lone pairs of O in OH form hydrogen bonds with surrounding water molecules and dimers, rather than forming conjugated orbitals with Fe (**panel c**). The remaining PDF peak located at 3.3 Å is also assigned to the Fe-Fe distance and probably indicates a deprotonated form, such as dioxo-bridged dimers (**panel d**).

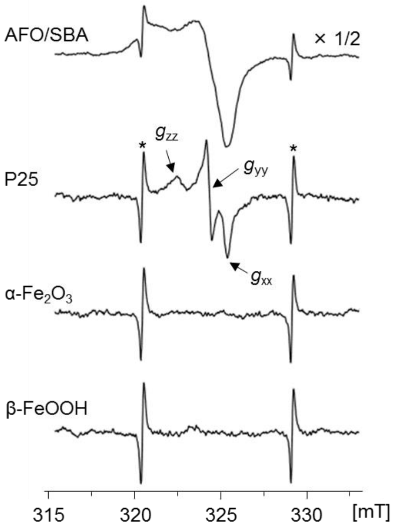

Fig. S10. Charge separation efficiency of AFO/SBA based on ESR data. AFO/SBA produces a considerably larger amount (*ca.* 10 times) of O_2^- than P25. Neither α-Fe_2O_3 nor β-FeOOH produced O_2^- under identical irradiation conditions. Asterisks indicate signals due to a standard marker, Mn^{2+}/MgO.

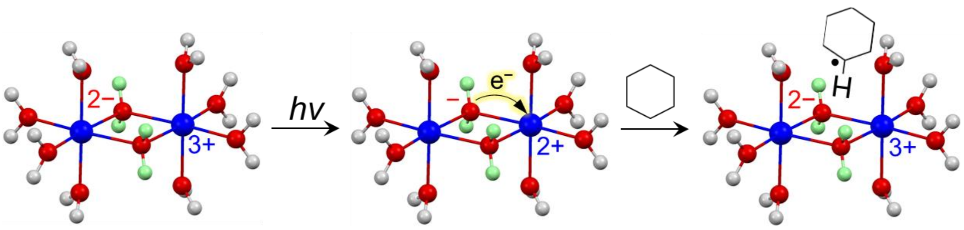

Fig. S11. Schematic representation for the mechanism of cyclohexane oxidation on ferric dimers.

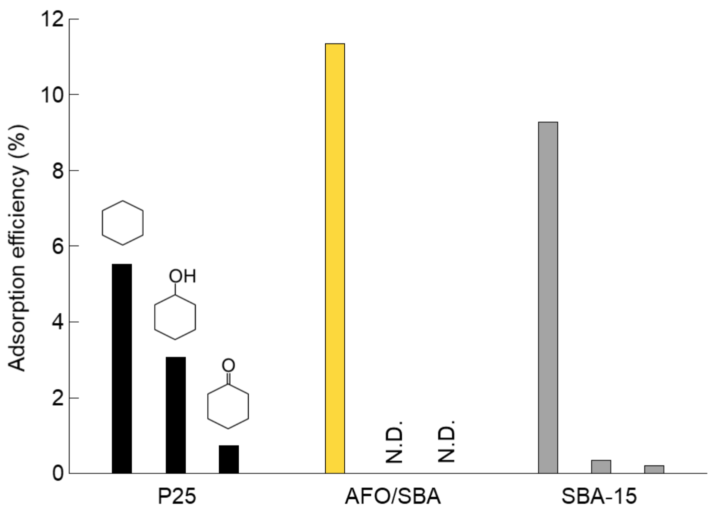

Fig. S12. Positive support effects of SBA-15 on the photocatalytic activity of AFO/SBA. Adsorption of cyclohexane, cyclohexanol and cyclohexanone on different materials from the mixture is tested. AFO/SBA adsorbs cyclohexane effectively and selectively.

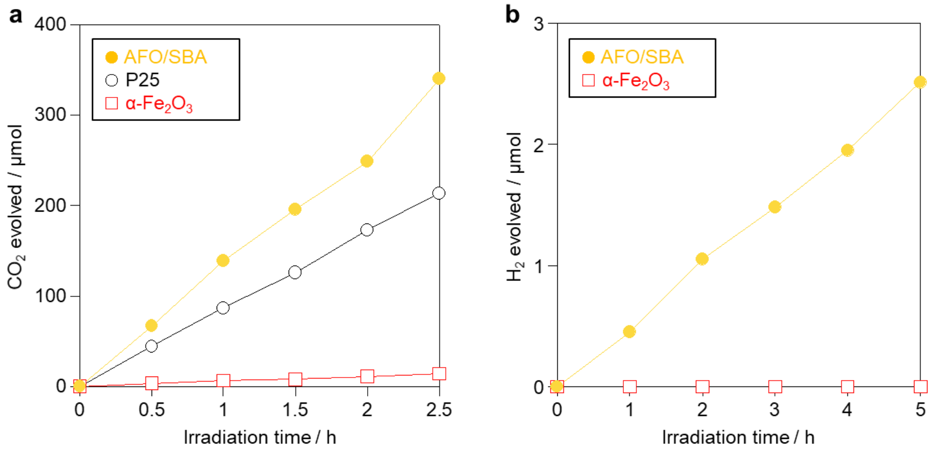

Fig. S13. Versatility of AFO/SBA photocatalyst. a, Time courses of CO_2 evolution from aerated aqueous solution of formic acid containing different photocatalysts under irradiation with solar light (λ > 300 nm). **b,** Time courses of H_2 evolution from deaerated aqueous solution containing different photocatalysts under irradiation with solar light (λ > 300 nm).

References

1. M. Zhu, B. W. Puls, C. Frandsen, J. D. Kubicki, H. Zhang, G. A. Waychunas, In situ structural characterization of ferric iron dimers in aqueous solutions: identification of μ-oxo species. *Inorg. Chem.* **52**, 6788-6797 (2013).
2. Michel, F. M., Barrón, V., Torrent, J., Morales, M. P., Serna, C. J., Boily, J.-F., Liu, G., Ambrosini, A., Cismasu, A. C. & Brown Jr., G. E. Ordered ferrimagnetic form of ferrihydrite reveals links among structure, composition, and magnetism. *Proc. Natl. Acad. Sci. USA* **107**, 2787-2792 (2010).
3. A. K. Soper, E. R. Barney, Extracting the pair distribution function from white-beam X-ray total scattering data. *J Appl Crystallogr* **44**, 714-726 (2011).
4. X. Wang, M. Zhu, L. K. Koopal, W. Li, W. Xu, F. Liu, J. Zhang, Q. Liu, X. Feng, D. L. Sparks, Effects of crystallite size on the structure and magnetism of ferrihydrite. *Environ. Sci.: Nano* 3, 190-202 (2016).
5. Weiss, A. & Choy, J. H. Phase changes in Fe(III)OCl−RNH_2-intercalation complexes. *Z. Naturforsch. B* **39**, 1193-1198 (1984).

Appendix. Structural information

(1) Ferrihydrite in FO/SBA obtained by the PDF curve fitting

*Note that the structure information was exported as $P1$ space group from the PDFgui program but the actual fitting was done under the symmetry constraint in the $P6_3mc$ space group.

(2) Hematite in FO/SBA obtained by the PDF curve fitting

*Note that the structure information was exported as $P1$ space group from the PDFgui program but the actual fitting was done under the symmetry constraint in the $H\bar{3}c$ space group.
